# FTO Alleviates CdCl_2_-Induced Apoptosis and Oxidative Stress via the AKT/Nrf2 Pathway in Bovine Granulosa Cells

**DOI:** 10.3390/ijms23094948

**Published:** 2022-04-29

**Authors:** He Ding, Zhiqiang Li, Xin Li, Xiaorui Yang, Jing Zhao, Jing Guo, Wenfa Lu, Hongyu Liu, Jun Wang

**Affiliations:** 1 Joint Laboratory of Modern Agricultural Technology International Cooperation, Ministry of Education, Jilin Agricultural University, Changchun 130118, China; dinghe1130@126.com (H.D.); lizhiqiangsky@126.com (Z.L.); lx13591909429@126.com (X.L.); yangxiaorui2020@163.com (X.Y.); jlndfztd@163.com (J.Z.); jguochn@163.com (J.G.); wenfa2004@163.com (W.L.); 2 Key Lab of Animal Production, Product Quality and Security, Ministry of Education, College of Animal Science and Technology, Jilin Agricultural University, Changchun 130118, China

**Keywords:** m^6^A, FTO, CdCl_2_, granulosa cells, apoptosis

## Abstract

Cadmium (Cd) is a common environmental heavy metal contaminant of reproduction toxicity. Cd accumulation in animals leads to the damage of granulosa cells. However, its mechanism needs to be elucidated. This research found that treating granulosa cells with Cd resulted in reduced cell viability. The flow cytometry results showed that Cd increased the degree of apoptosis and level of superoxide anion (O_2_^−^) in granulosa cells. Further analysis showed that Cd treatment resulted in reduced expression levels of nuclear factor erythroid 2-related factor-2 (Nrf2), superoxide dismutase (SOD), catalase (CAT) and NAD(P)H: quinone oxidoreductase 1 (NQO1), and an increased expression level of malondialdehyde (MDA); the expression levels of Bcl-2 associated X (Bax) and caspase-3 increased, whereas that of B-cell lymphoma 2 (Bcl-2) decreased. Changes in m^6^A methylation-related enzymes were noted with Cd-induced damage to granulosa cells. The results of transcriptome and MeRIP sequencing revealed that the AKT pathway participated in Cd-induced damage in granulosa cells, and the MAX network transcriptional repressor (*MNT*) may be a potential target gene of fat mass and obesity-associated protein (FTO). FTO and YTH domain family member 2 (YTHDF2) regulated *MNT* expression through m^6^A modification. FTO overexpression alleviated Cd-induced apoptosis and oxidative stress through the activation of the AKT/Nrf2 pathway; this process could be reversed using siMNT. Overall, these findings associated m^6^A with Cd-induced damage to granulosa cells and provided insights into Cd-induced granulosa cell cytotoxicity from a new perspective centered on m^6^A modification.

## 1. Introduction

The development and maturation of oocytes determine the reproductive ability of female animals. Granulosa cells supply necessary nutrition and small molecules for the oocytes through gap junctions, which is crucial for oocyte maturation [1]. With the acceleration of global industrialization, Cd pollution has gradually spread worldwide. Pollution due to the heavy metal cadmium is particularly severe in developing countries. Several studies have focused on cadmium pollution; some of these studies have explored the alterations in the aquatic environment in Malaysia, agricultural soil in China, and water and food qualities in northern Vietnam with higher cadmium concentrations [2,3,4]. Various industrial activities lead to heavy metal-induced environmental pollution, including water, soil, and plant pollution; the heavy metals in soil and animal feed may accumulate into animal yields. The cadmium content in milk in industrial areas is significantly higher than that in unpolluted areas; the cadmium in milk may come from the soil in the following manner: from soil to silage to milk [5]. A significantly positive correlation has been reported between Cd concentration in cattle tissues and their feed [6]. Cadmium can accumulate in animal tissues, including ovaries, and endanger reproductive functions [7,8,9,10]. Studies have shown that granulosa cells may be one of the targets of Cd toxicity. Cd treatment can affect progesterone secretion in granulosa cells [11], thus increasing follicular atresia and apoptosis [12]. The use of cadmium chloride (CdCl_2_) to treat porcine granulosa cells demonstrated that Cd can increase reactive oxygen species (ROS) levels, and the degree of apoptosis increases in a dose- and time-dependent manner [13].

The Myc/Max/Mad network is involved in the regulation of various biological processes, including cell proliferation and apoptosis. Myc/Max and Mad/Max dimers play transcriptional activation and transcriptional repression effects, respectively, by binding to the E-box region of target genes. MNT is the most special member of the Mad family because it is the largest protein in the family and is widely expressed in human tissues, participating in a variety of biological processes [14]. MNT has an important role in development, and the deletion of MNT causes the death of mice shortly after birth [15,16]. MNT shares a basic helix-loop-helix-leucine zipper (bHLHLZ) domain and a SIN3-interacting domain (SID). bHLHLZ is used to interact with Max and bind the E-box of DNA, while SID is required for the inhibitory function of MNT [17,18]. Myc can induce apoptosis by inhibiting the expression of anti-apoptotic genes Bcl-X and Bcl-2. Mad/Max can compete with Myc/Max to bind to E-box and regulate the expression of target genes [19].

N6-methyladenosine (m^6^A) modification is widely present in various tissues and organs of animals and results in dynamic and reversible changes in various tissues across the developmental stages [20,21]. It mainly occurs in the consensus sequence 5’-RRACH-3’ (R = A or G, G > A; H = A, C, or U, U > A > C) and is highly conserved in several species [22,23,24,25]. The research map shows that m^6^A mainly occurs in the vicinity of stop codons and 3ʹ untranslated regions (3ʹUTRs) [26]. The m^6^A is influenced by multiple effectors, including the writer, eraser, and reader. The “writer”, as methyltransferases, mainly include methyltransferase-like protein 3 (METTL3), methyltransferase-like protein 14 (METTL14), and Wilms tumor 1-associating protein (WTAP). The “erasers”, as demethylases, mainly include AlkB Homolog 5 (ALKBH5) and fat mass and obesity-associated protein (FTO) [27]. The “readers”, as binding proteins, mainly include YTH domain-containing protein 1−2 (YTHDC1-2) and YTH domain family member 1−3 (YTHDF1-3). The reader can recognize m^6^A modifications and regulate the translation, shearing, transportation, positioning, and stability of RNA at the posttranscriptional level to ensure that m^6^A can perform its biological functions [26,28].

As a demethylase, FTO can reduce the m^6^A modification level of target genes. FTO is involved in a variety of biological processes and cell death. Overexpression of FTO inhibited the expression of pro-apoptotic proteins caspase-3, caspase-9 and Bax in vivo and in vitro experiments, thereby inhibiting mitochondria-dependent apoptosis in adipocytes [29]. FTO functions by recognizing m^6^A modification, and the inhibition of the FTO-induced m^6^A modification level may be related to MEHP-induced Leydig cell apoptosis [30]. FTO is involved in cell death, induced by a variety of toxicants. Cobalt is an environmental toxicant that can damage human health. CoCl_2_ induces the production of ROS in human neuroglioma H4 cells and affects the m^6^A modification level of apoptosis-related genes by reducing the expression of FTO, resulting in the activation of apoptosis [31]. It has been shown that the demethylase FTO can decrease the level of m^6^A on *flotillin 2* (*FLOT2*) mRNA, increase the stability of *FLOT2* mRNA, and induce the dysfunction of granulosa cells [32]. In human granulosa cells, FTO knockdown decelerates the decomposition of *FOS*-mRNA and upregulates FOS levels, ultimately leading to GC-mediated ovarian aging [33]. Several studies have reported that m^6^A methylation-related enzymes affect apoptosis in various cells, including those of the ovaries and testes [34,35,36].

However, the role of RNA m^6^A modification in Cd-induced apoptosis and oxidative stress in bovine ovarian granulosa cells remains to be investigated. Therefore, this study investigated how the m^6^A protects granulosa cells from Cd-induced apoptosis.

## 2. Results

### 2.1. Cd Increased the Degree of Apoptosis in Granulosa Cells

**Figure 1:**
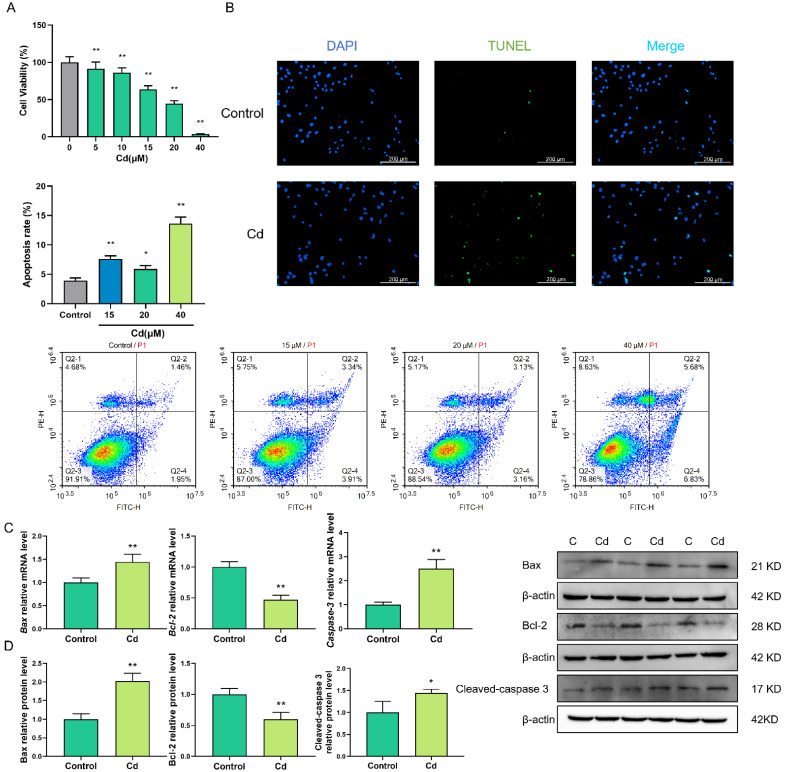
Cd increased the degree of apoptosis in granulosa cells. (**A**) Analysis of the viability of cells exposed to Cd using CCK-8. (**B**) Flow cytometry analysis of the degree of apoptosis in the control and Cd groups. The TUNEL assay detected apoptotic cells in the control and Cd groups. Green fluorescence represents apoptotic cells. (**C**) RT-qPCR results revealed the transcript levels of these genes after Cd treatment. (**D**) Protein levels after Cd treatment were analyzed by Western blotting. * *p* < 0.05; ** *p* < 0.01.

The viability of granulosa cells treated with Cd was assessed by CCK-8 (Figure 1A). Compared with the control group, 5 μM of Cd significantly reduced cell viability (*p* < 0.01), and cell viability decreased with an increasing Cd concentration. Flow cytometry analysis results indicated that 15−40 μM of Cd significantly increased the degree of apoptosis of the granulosa cells (*p* < 0.05; Figure 1B). A concentration of 15 μM was used as the subsequent treatment concentration. The TUNEL results indicated that the proportion of TUNEL-positive cells increased markedly after Cd treatment (Figure 1B). Subsequently, RT-qPCR and a Western assay indicated that Bax and caspase-3 expressions were significantly increased and Bcl-2 expression was significantly reduced after Cd treatment (*p* < 0.05; Figure 1C,D). In conclusion, Cd induces apoptosis in granulosa cells.

### 2.2. Cd-Induced Oxidative Stress in Granulosa Cells

**Figure 2:**
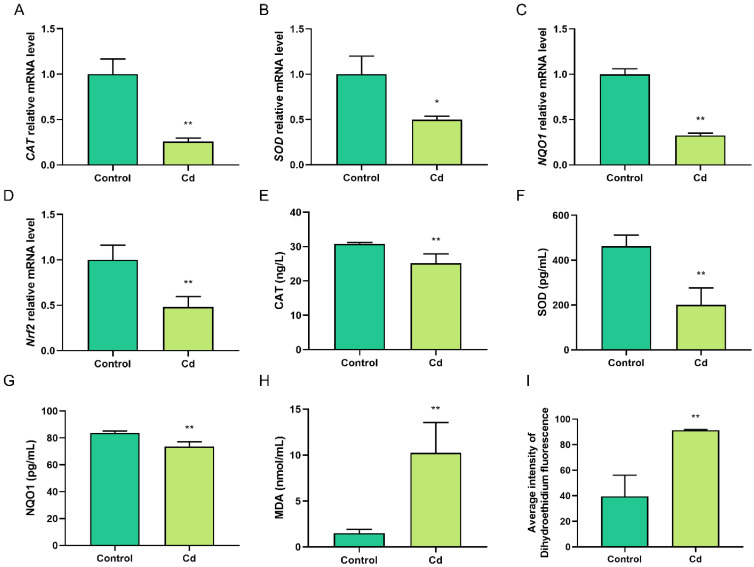

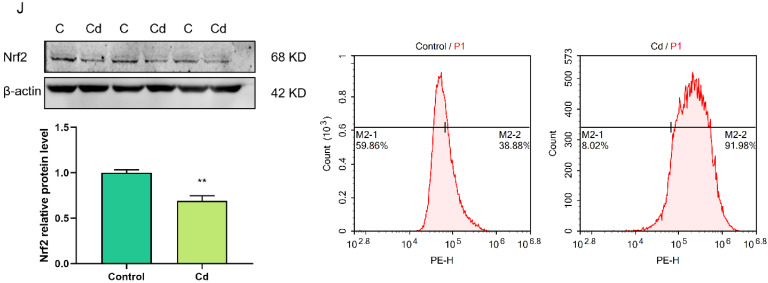
Cd-induced oxidative stress in granulosa cells. (**A**–**C**) RT-qPCR results revealed the transcript levels of *CAT*, *SOD*, and *NQO1* after Cd treatment. (**D**) RT-qPCR results revealed the transcript levels of *Nrf2* after Cd treatment. (**E**–**H**) ELISA results revealed the intracellular levels of CAT, SOD, NQO1, and MDA after Cd treatment. (**I**) Intracellular reactive oxygen species (ROS) was measured using flow cytometry analysis (DHE) in the control and Cd groups. (**J**) Nrf2 protein levels were analyzed by Western blotting after Cd treatment. * *p* < 0.05; ** *p* < 0.01.

CAT, SOD, and NQO1 are the key components of the antioxidant gene, and the RT-qPCR analysis showed that the transcriptional levels of *CAT*, *SOD*, and *NQO1* decreased following Cd treatment (*p* < 0.05; Figure 2A–C). In addition, the intracellular CAT, SOD, and NQO1 levels decreased significantly, which was accompanied by an increase in the MDA levels (*p* < 0.01; Figure 2E–H). Intracellular reactive oxygen species (ROS) was measured using flow cytometry analysis (DHE), and fluorescence intensity increased in the Cd group (*p* < 0.01; Figure 2I). Further testing found that the protein and mRNA expression of Nrf2 decreased significantly with Cd treatment (*p* < 0.01; Figure 2D,J). The above results suggested that Cd treatment leads to decreased antioxidant levels and increased ROS levels in granulosa cells, which induced oxidative stress.

### 2.3. Cd Alters the Expression of m^6^A Methylation-Regulated Genes in Granulosa Cells

**Figure 3:**
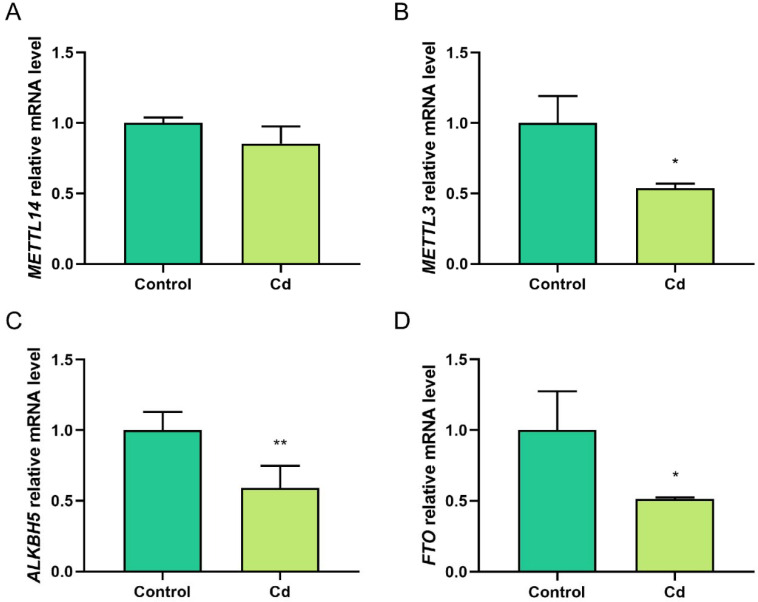
Expression of m^6^A methylation-regulated genes in Cd-treated granulosa cells. (**A**–**D**) RT-qPCR results revealed transcript levels of *METTL14. METTL3*, *ALKBH5*, and *FTO* after Cd treatment. * *p* < 0.05; ** *p* < 0.01.

We conducted a determination of mRNA expression levels of essential genes that regulate m^6^A modification after Cd treatment by RT-qPCR. The results indicated that *METTL3*, *ALKBH5*, and *FTO* were significantly downregulated after Cd treatment (*p* < 0.05; Figure 3A–D). 

### 2.4. Transcriptome and MeRIP Sequencing to Identified m^6^A modification Targets

**Figure 4:**
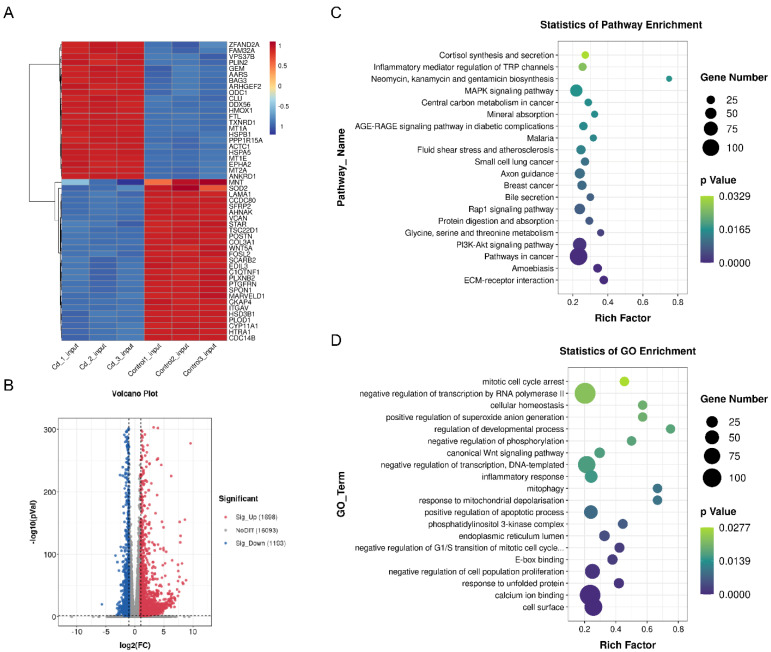

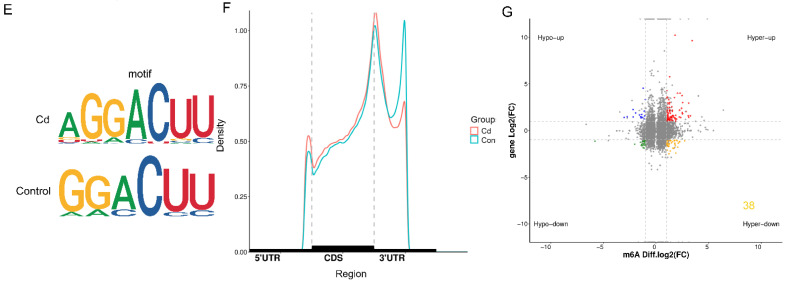
Transcriptome and MeRIP sequencing of the control and Cd groups. (**A**) Heat map results revealed differential transcript levels after Cd treatment. (**B**) Volcano map showed the differentially expressed mRNAs in the control and Cd groups. (**C**,**D**) GO and KEGG analyses results revealed the enrichment of differential genes in the control and Cd groups. (**E**) The HOMER motif analysis results revealed the m^6^A motif in granulosa cells. (**F**) Density distribution of m^6^A enrichment across mRNA transcriptome in granulosa cells. (**G**) Distribution of peaks with significant differences in the RNA expression and m^6^A modification level in the control and Cd groups.

Differentially expressed genes were screened out via transcriptome sequencing under the conditions of |log_2_(FC)| of ≥1 and a *p*-value of <0.05. The heat and volcano map revealed significant differences between the two groups, and 2891 genes were screened for differential expression, 1698 of which were upregulated and 1193 were downregulated (Figure 4A,B). The different genes in the control and Cd groups were analyzed through GO and KEGG analyses. Under the standard of *p* < 0.05, 20 GO function enrichment and 20 KEGG pathways were screened out (Figure 4C,D). In the control and Cd groups, phosphatidylinositol 3-kinase complex (GO:0005942) and PI3K-Akt signaling pathway (ko04151) were enriched. In addition, positive regulations of O_2_^−^ generation (GO:0032930) and apoptotic process (GO:0043065) were significantly enriched. MeRIP sequencing results confirmed that the m^6^A consensus motif was GGAC (Figure 4E). These methylation sites were primarily enriched in stop codons and 3′UTRs (Figure 4F). Finally, in the control and Cd groups, 165 significantly different genes from MeRIP-seq and mRNA-seq sequencing were screened out, of which 38 genes were upregulated in m^6^A modification and downregulated in mRNA expression (Figure 4G).

### 2.5. FTO Affects m^6^A Modification and Expression Level of the MNT mRNA

**Figure 5:**
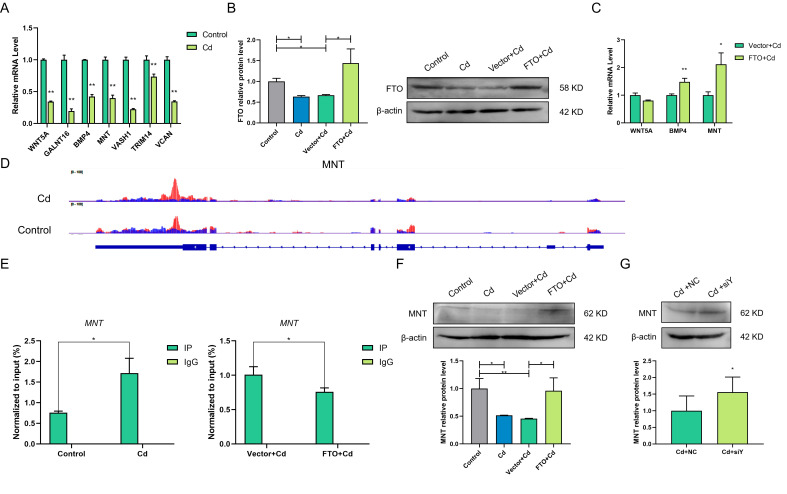
FTO affects m^6^A modification and the expression level of the *MNT* mRNA. (**A**) RT-qPCR results revealed the relative expression of seven potential target genes in the control and Cd groups. (**B**) Determination of FTO expression level after transfection with pcDNA3.1-FTO or pcDNA3.1-Vector. (**C**) RT-qPCR results revealed the mRNA expression of target genes after FTO overexpression. (**D**) Distribution of m^6^A peaks across *MNT* transcriptome. (**E**) Determination of m^6^A modification level of *MNT* mRNA after Cd treatment and FTO overexpression using MeRIP-RT-qPCR. (**F**) Western blotting results showed MNT protein expression after Cd treatment and FTO overexpression. (**G**) Western blotting results showed MNT protein expression after Cd and siYTHDF2 treatments. * *p* < 0.05; ** *p* < 0.01.

Based on the sequencing results, we preferentially screened seven highly expressed genes related to apoptosis from the hyper-down group (Figure 4G) for further verification. The results showed that the potential targets were significantly decreased after Cd treatment (Figure 5A). Among them, are *WNT5A* (GO:0043066, negative regulation of the apoptotic process), *BMP4* (GO:0043066, negative regulation of the apoptotic process), and *MNT* (GO:2001234, negative regulation of the apoptotic signaling pathway) were involved in the negative apoptosis regulation. These genes were most likely FTO target genes. FTO overexpression treatment was performed, and FTO was highly expressed after transfection with pcDNA3.1-FTO (*p* < 0.05; Supplementary Figure S1 and Figure 5B). The results indicated that FTO overexpression increased *BMP4* and *MNT* mRNA levels significantly (*p* < 0.05; Figure 5C). The distribution of m^6^A peaks of MNT was analyzed; the m^6^A modification of *MNT* increased after Cd treatment (Figure 5D). MeRIP-RT-qPCR results revealed that m^6^A modification of *MNT* was significantly increased after Cd treatment and it was significantly decreased when FTO was overexpressed (*p* < 0.05; Figure 5E), identifying *MNT* as a potential target of FTO. Next, Western blot results confirmed that FTO upregulated the Cd-induced decrease in MNT expression (*p* < 0.05; Figure 5F). YTHDF2 can recognize m^6^A modification and degrade target genes. We treated cells with siTYHDF2 (*p* < 0.05; Supplementary Figure S2) and found that a decreased YTHDF2 expression increased MNT expression (*p* < 0.05; Figure 5G).

### 2.6. FTO Overexpression Suppressed Cd-Induced Apoptosis, Perhaps by Activating the AKT Pathway in Granulosa Cells

**Figure 6:**
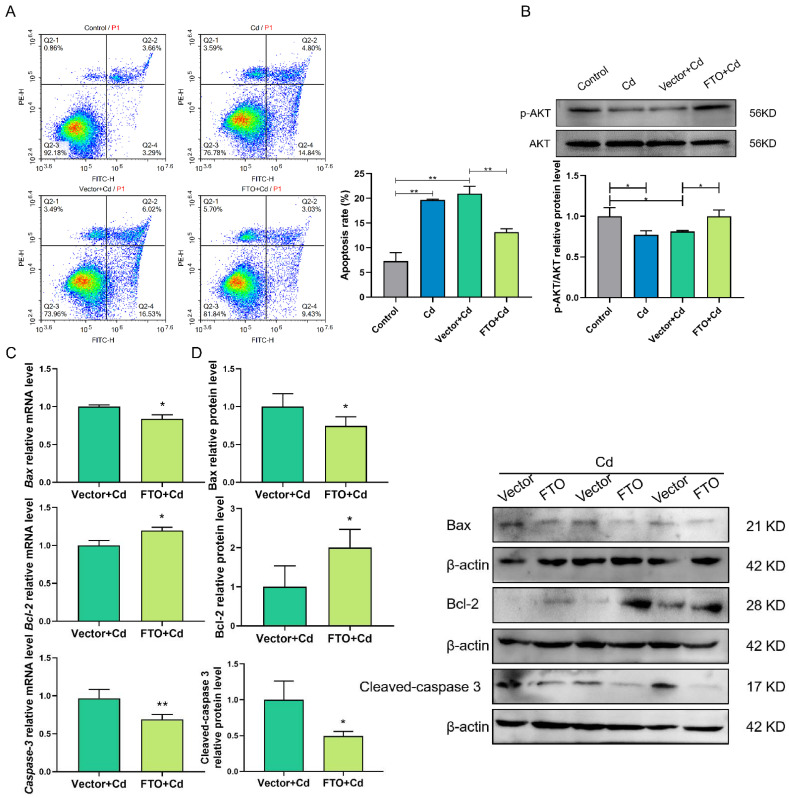
FTO overexpression suppressed Cd-induced apoptosis, perhaps by activating the AKT pathway in granulosa cells. (**A**) Flow cytometry analysis results detected apoptosis rate in the control, Cd, Vector + Cd, and FTO + Cd groups. (**B**) The p-AKT protein levels were analyzed by Western blotting after Cd and FTO treatments. (**C**,**D**) RT-qPCR and Western blotting results indicated the mRNA and protein levels of Bax, Bcl-2, and caspase-3 after FTO overexpression. * *p* < 0.05; ** *p* < 0.01.

The role of FTO on Cd-induced apoptosis was researched. The degree of apoptosis assay showed that FTO overexpression suppressed Cd-induced apoptosis in granulosa cells (*p* < 0.01; Figure 6A). Further analysis showed that FTO overexpression reduced the expression levels of Bax and caspase-3 and increased that of Bcl-2 (*p* < 0.05; Figure 6C,D). Investigation of the associated pathways indicated that Cd significantly reduced the protein expression of p-AKT and FTO overexpression reversed this phenomenon (*p* < 0.05; Figure 6B). In short, the AKT pathway participates in the protective function of FTO on Cd-induced damage of granulosa cells.

### 2.7. FTO Overexpression Suppressed Cd-Induced Oxidative Stress

**Figure 7:**
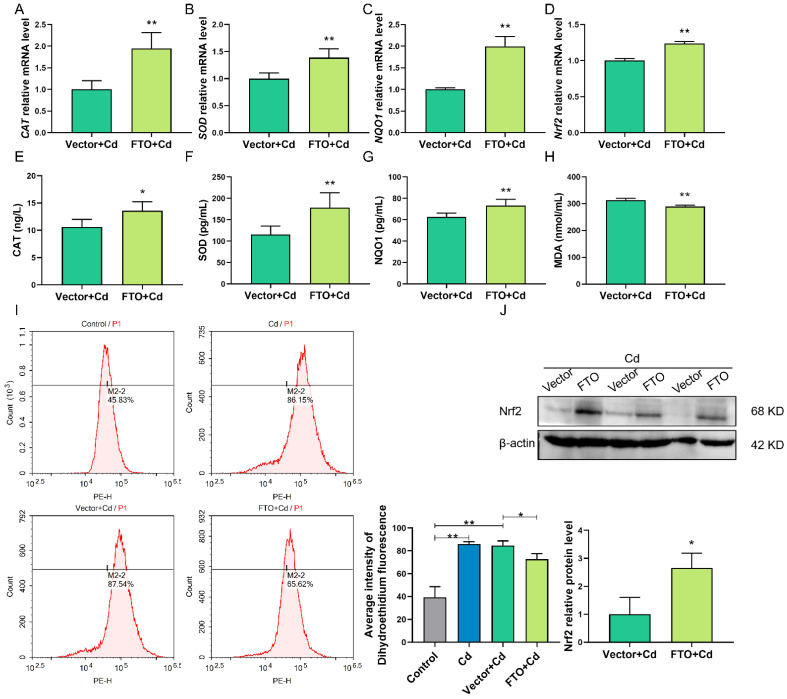
FTO overexpression suppressed Cd-induced oxidative stress. (**A**–**D**) RT-qPCR revealed the mRNA levels of *CAT*, *SOD*, *NQO1*, and *Nrf2* after Cd and FTO treatments. (**E**–**H**) ELISA results showed the intracellular levels of CAT, SOD, NQO1, and MDA after Cd and FTO treatments. (**I**) Intracellular ROS level was measured using flow cytometry analysis (DHE) in the control, Cd, Vector + Cd, and FTO + Cd groups. (**J**) Nrf2 protein levels were analyzed by Western blotting after Cd and FTO treatments. * *p* < 0.05; ** *p* < 0.01.

The role of FTO in Cd-induced oxidative stress was assessed. RT-qPCR indicated that increasing FTO expression increased the transcriptional levels of *CAT*, *SOD*, and *NQO1* (*p* < 0.01; Figure 7A–C) and significantly increased the intracellular levels of CAT, SOD, and NQO1, which was accompanied by a decrease in the level of MDA (*p* < 0.05; Figure 7E–H). Further analysis using flow cytometry analysis (DHE) showed that FTO overexpression reduced the levels of intracellular ROS (*p* < 0.05; Figure 7I). These might be because of FTO overexpression, which increased the expression of Nrf2 mRNA and protein (*p* < 0.05; Figure 7D,J).

### 2.8. siMNT Reverses the Protective Effect of FTO during Cd Injury in Granulosa Cells

**Figure 8:**
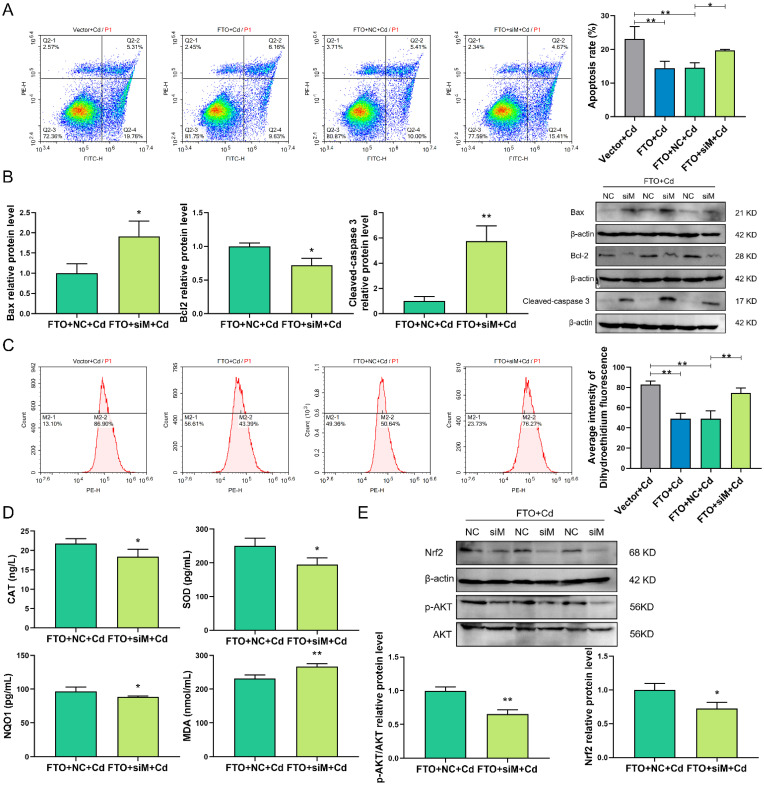
siMNT reverses the protective effect of FTO during Cd injury in granulosa cells. (**A**) Determination of the degree of apoptosis in the Vector + Cd, FTO + Cd, FTO + NC + Cd, and FTO + siM + Cd groups using flow cytometry analysis. (**B**) Western blotting results showed the protein expression levels of Bax, Bcl-2, and cleaved caspase-3 after siMNT and FTO treatments under Cd injury. (**C**) Intracellular level of ROS was measured using flow cytometry analysis (DHE) in Vector + Cd, FTO + Cd, FTO + NC + Cd, and FTO + siM + Cd groups. (**D**) ELISA results show the intracellular levels of CAT, SOD, NQO1, and MDA. (E) Nrf2 and AKT protein levels were analyzed by Western blotting after siMNT and FTO treatments under Cd injury. * *p* < 0.05; ** *p* < 0.01.

To explore the role of MNT in FTO protection of granulosa cells from Cd-induced damage, siMNT was transfected into granulosa cells co-treated with FTO and Cd. The interference efficiency of *MNT* is indicated in Supplementary Figure S3. In addition, FTO decreased Cd-induced apoptosis of granulosa cells, whereas the interference with MNT attenuated the effect of FTO (*p* < 0.05; Figure 8A). Similarly, interfering with MNT reduced the protein level of Bcl-2 and increased that of cleaved caspase-3 and Bax (*p* < 0.05; Figure 8B). Additionally, MNT was involved in protecting FTO against Cd-induced oxidative damage. Flow cytometry analysis (DHE) revealed that MNT interference leads to increased intracellular levels of ROS (*p* < 0.01; Figure 8C). In the MNT interference group, intracellular levels of CAT, SOD, and NQO1 were also significantly reduced, which was accompanied by an increase in the level of MDA (*p* < 0.05; Figure 8D). In detecting the Nrf2 and AKT pathways, it was found that MNT knockdown resulted in the downregulation of the protein levels of Nrf2 and AKT (*p* < 0.05; Figure 8E), which might be the reason why the protective effect of FTO on Cd-induced damage of granulosa cells was weakened after MNT interference.

## 3. Discussion

As far as we know, the effect of Cd on the apoptosis and oxidative stress of bovine ovarian granulosa cells has not yet been reported. Our study indicated that m^6^A modification is involved in Cd-induced granulosa cell apoptosis and oxidative stress and identified FTO as a crucial factor affecting this process. In Cd injury, FTO regulates MNT expression through the m^6^A modification pathway. Additionally, FTO overexpression attenuates Cd-induced apoptosis and oxidative stress and activates the AKT and Nrf2 pathways, which were attenuated after MNT interference. In short, Cd modulates the expression of MNT in an m^6^A-modified manner, affecting apoptosis and oxidative stress in granulosa cells through the AKT/Nrf2 pathway.

Cd is reproductively toxic and affects the reproductive performance of animals. Several studies have reported the effect of Cd on germ cells. In the human granulosa cell line (KGN), Cd induces apoptosis in a dose- and time-dependent manner, and is accompanied by changes in the expression levels of Bax, BAK and Bcl-2. Cd may increase the intracellular ROS and Ca^2+^ levels and reduce mitochondrial membrane potential, thereby inducing oxidative stress in KGN cells [37]. Moreover, reported in chicken ovarian granulosa cells, Cd increases ROS production and apoptosis [38]. Cd also enhances ROS production and induces apoptosis in many other cells, including the thymus, hepatocytes, and leydig TM3 cells [39,40,41]. These findings are in line with our research, which revealed that Cd decreases the viability of granulosa cells in a concentration-dependent manner. Cd treatment promotes apoptosis, increases Bax and caspase-3 expression levels, and decreases Bcl-2 expression levels. Moreover, Cd induces oxidative stress in granulosa cells; particularly, the expression levels of CAT, SOD, and NQO1 decreased and those of MDA and O_2_^−^ increased. In our study, Cd-induced damage of granulosa cells resulted in changes in m^6^A methylases, including the demethylase FTO. Previous studies have also reported that Cd leads to changes in the level of m^6^A modification [42,43], and FTO is involved in regulating ovarian aging and granulosa cell dysfunction [32,33].

The m^6^A could be participating in regulating Cd-induced damage in granulosa cells. Therefore, further analysis was performed through transcriptome and MeRIP sequencing. MeRIP sequencing indicated that the m^6^A consensus motif was consistent with 5’-RRACH-3’. Through the combined analysis of transcriptome and MeRIP sequencing, a potential target gene *MNT* was screened, which acts as a MAX network transcriptional repressor and is involved in the negative regulation of the apoptotic signaling pathway. MNT proteins use MAX as a cofactor for DNA binding and both MNT and MAX as a complex for the transcriptional repression [14]. MNT can also work independently, and it is highly expressed in the human ovary. MNT is associated with various cellular processes, including apoptosis [14]. MNT is expressed in the granulosa cell, and the lack thereof leads to embryonic growth defects and death in mice [15]. The deletion of MNT promotes apoptosis in mouse embryo fibroblasts [16]. The reduction in MNT expression enhances the induction of apoptosis by lithocholic acid in primary human hepatocytes [44]. In addition, the deletion of MNT promotes ROS generation [45]. Our results indicated that Cd increases the m^6^A modification level of *MNT* mRNA and decreases MNT expression, whereas siYTHDF2 increases MNT expression, which may be because YTHDF2 recognizes m^6^A modification to degrade *MNT* mRNA. Previous evidence shows that YTHDF2 recognizes m^6^A-modified degradation target genes [46]. Cd-induced reduction in the MNT level in an m^6^A-dependent manner may be responsible for granulosa cell damage. When FTO was overexpressed, the level of m^6^A modification of *MNT* mRNA decreased, whereas the expression level of MNT protein increased. These results suggest that, in Cd-treated granulosa cells, MNT expression is regulated via m^6^A modification involved in Cd injury. To our knowledge, this research is the first to explore the function of MNT in granulosa cell damage.

Transcriptome sequencing revealed that the PI3K-Akt signaling pathway was enriched in KEGG and GO analyses after Cd treatment, suggesting that the PI3K-Akt pathway participated in Cd-induced damage of granulosa cells. A study regarding the effect of Cd on germ cells found that the PI3K/Akt/Nrf2 pathway is involved in Cd-induced cell damage [13,47], and the PI3K-Akt pathway has an essential role in cell survival and regulates apoptosis by MNT [48]. These findings are consistent with our findings obtained from this study. In addition, positive regulation of O_2_^−^ generation was significantly enriched in the GO analysis, and we confirmed that Cd treatment leads to an increased level of O_2_^−^.

Furthermore, m^6^A methylation is involved in the regulation of apoptosis and oxidative stress. Our results showed that FTO overexpression decreased Cd-induced granulosa cell apoptosis and oxidative stress; the AKT/Nrf2 pathway is activated in this process. siMNT partially reversed the effect of FTO. Although an AKT inhibitor was not used in this study, it is well known that the AKT/Nrf2 pathway is involved in anti-apoptotic and oxidative stress processes. As noted in previous studies, AKT/Nrf2 inhibited granulosa cell apoptosis and oxidative stress in different treatments [49,50,51,52]. The effect of m^6^A on the AKT/Nrf2 pathway has been studied previously. In ovarian cancer cells, FTO overexpression significantly reduced apoptosis and promoted AKT phosphorylation. FTO silencing exhibited contrasting results [34]. Another study found that the environmental endocrine disruptor di(2-ethylhexyl) phthalate leads to testicular damage in rats, inhibits the antioxidant function of Nrf2, and increases apoptosis and oxidative stress, which is accompanied by an increased level of m^6^A modification of the Nrf2 mRNA and decreased level of FTO [35]. The above studies suggest the reliability of our results. FTO may play a role in protecting granulosa cells from Cd-induced damage by regulating MNT expression and AKT/Nrf2 activity through m^6^A modification. Heavy metal poisoning is a chronic and long-term process, and the reproductive toxicity associated with Cd pollution should be further verified using in vivo experiments.

## 4. Materials and Methods

### 4.1. Primary Cell Culture

The ovaries of healthy cattle were collected from a slaughterhouse, stored in a container with 37 °C normal saline, and sent to the study laboratory within 4 h. The ovaries were washed five times with normal saline containing penicillin/streptomycin (Sangon Biotech, Shanghai, China) and were immediately transferred to the cell culture room for extraction. We used a 5 mL syringe to aspirate the follicular fluid from 3–8 mm follicles, centrifuged at 1000 rpm for 5 min to collect the cells, then washed with PBS containing penicillin/streptomycin for 2–3 times, and centrifuged again to collect the cells. Granulosa cells were cultured in DMEM/F-12 (Gibco, Waltham, MA, USA). The seeding density was 2 × 10^5^ cells/well. The granulosa cells were cultured at 37 °C in the presence of 5% CO_2_. Granulosa cells were processed with 5, 10, 15, 20, and 40 μM of CdCl_2_ (MACKLIN, Shanghai, China). At least 40 ovaries were used at a time for the analysis.

### 4.2. Cell Viability

Granulosa cells were cultured in 96-well plates (2 × 10^4^ cells/well) at 37 °C in the presence of 5% CO_2_ for 24 h. The cells were treated with 5, 10, 15, 20, and 40 μM of CdCl_2_ for 24 h, with six replicate wells for each concentration. Then, 10 μL of CCK-8 (APEXBIO, Houston, TX, USA) solution was added and incubated for 4 h in the dark. A microplate reader (BioTek, Winooski, VT, USA) was used to determine the absorbance at 450 nm.

### 4.3. Transient Cell Transfection

Transfection was performed when the granulosa cells reached 70% confluence. The siRNAs (Supplementary Table S1) were produced by GenePharma (GenePharma Biotechnology, Shanghai, China). The procedure was as previously described [53]. In short, to prepare the transfection solution for each group, 200 μL of DMEM/F-12 was used to dilute 9 μL of Lipofectamine™ 2000 and 3 μg of pcDNA3.1-FTO or pcDNA3.1-Vector and incubated for 5 min at room temperature. Subsequently, Lipofectamine™ 2000 and pcDNA3.1-FTO or pcDNA3.1-Vector dilution were mixed and incubated for 20 min at room temperature. The cells were washed twice or thrice with PBS, transferred to a 1.6 mL serum-free culture medium, and incubated for 6 h at 37 °C in the presence of 5% CO_2_. The complete culture medium was replaced to continue culture for 18 h. The amounts of silencing MNT (siMNT) and siYTHDF2 used were 200 pmol for both, and the operation steps were similar to the abovementioned steps. The cultured cells were obtained for subsequent experiments.

### 4.4. Real-Time Quantitative PCR (RT-qPCR)

Total RNA from the granulosa cells was extracted using TRIzol (Takara, Tokyo, Japan). The steps for RNA reverse transcription and RT-qPCR were the same as before [53]. PrimeScript™ RT reagent Kit (Takara, Tokyo, Japan) and SYBR^®^ Premix Ex Taq™ II (Takara, Tokyo, Japan) for reverse transcription and RT-qPCR processes were used, respectively. The relative gene expression was analyzed by the 2^−∆∆Ct^ comparison method. Primer sequences are provided in Supplementary Table S2.

### 4.5. Western Blot Analysis

Cells were lysed in RIPA buffer (Beyotime, Shanghai, China), and then centrifuged at 12,000 rpm for 15 min at 4 °C. The proteins were separated by 10–12% SDS-PAGE and transferred to nitrocellulose membranes (Merck Millipore, Darmstadt, Germany). After blocking with Intercept PBS blocking buffer (LI-COR Biosciences, Lincoln, NE, USA) for 1.5 h, they were incubated with the primary antibodies at 4 °C overnight. After washing with TBST (5 × 5 min), the secondary antibody was incubated for 1 h. ECL (NCM Biotech, Suzhou, China) was used for chemiluminescence detection. The antibodies are provided in Supplementary Table S3.

### 4.6. Apoptosis Assay

Cd-treated cells in dishes were collected with trypsin and resuspended in 200 μL of 1X buffer containing FITC-Annexin V (5 μL) and PI (5 μL) (BD Biosciences, San Jose, CA, USA) for staining. Then, incubated at 37 °C for 15 min in the dark. Subsequently, 200 μL of 1X buffer was added to each tube. Approximately 30,000 cells were examined for each sample. The apoptosis was determined by a flow cytometer (FITC and PerCP channels) (ACEA Biosciences, Hangzhou, China).

### 4.7. TUNEL Assay

Cells were fixed with 4% paraformaldehyde for 20 min, permeabilized with 0.2% Triton X-100, incubated with TUNEL reagent at 37 °C for 1 h, and stained with DAPI for 10 min (Beyotime, Shanghai, China). Finally, Gene5 software was used to acquire the images of apoptotic cells.

### 4.8. ELISA

After Cd treatment, the cells were disrupted by repeated freezing and thawing to obtain an intracellular suspension. They were centrifuged at 3000 rpm for 20 min at 4 °C, and the supernatant was collected carefully. The intracellular components were analyzed by ELISA kit (MEIMIAN, Jiangsu, China), following the producer’s instructions.

### 4.9. ROS Assay

Following the manufacturer’s instructions, detection of superoxide anion (O_2_^−^) in granulosa cells by dihydroethidium (DHE) fluorescent probe occurred (Beyotime, Shanghai, China). The procedure was as follows. The DHE powder was dissolved in DMSO to prepare a stock solution at a concentration of 10 mM. The cells were collected into a 1.5 mL centrifuge tube, DHE stock solution at a ratio of 1:1000 was added, and the final concentration of DHE was 10 μM. Incubate at 37 °C for 30 min, and then wash the cells with PBS 2–3 times for ROS detection.

### 4.10. Transcriptome Sequencing and MeRIP Sequencing Assay

This method is similar to the previous study in [30]. Briefly, total RNA from the granulosa cells was extracted using TRIzol (Invitrogen, Carlsbad, CA, USA). RNA quality and quantity were determined by NanoDrop ND-1000 (NanoDrop, Wilmington, DE, USA) and Bioanalyzer 2100 (Agilent, Santa Clara, CA, USA), and a RIN number of >7.0. Purify Poly(A) RNA was purified from 50 μg of total RNA by Dynabeads Oligo (dT)25-61005 (Thermo Fisher, Lake Success, NY, USA) and then was fragmented into small pieces using the Magnesium RNA Fragmentation Module (NEB, Ipswich, MA, USA). Subsequently, the fragmented RNA was incubated for 2 h at 4 °C with an m^6^A antibody (No. 202003, Synaptic Systems, Gottingen, SN, Germany). The above products were reverse transcribed, and further screened and purified to construct a cDNA library with a fragment size of 300 bp ± 50 bp. Finally, 2 × 150 bp paired-end sequencing was performed using the Illumina Novaseq™ 6000 (LC-Bio, Hangzhou, China) according to the vendor’s protocol.

### 4.11. Bioinformatics

This method is similar to the previous study in [54]. Reads containing adapter contamination, low-quality bases, and undetermined bases were removed using the Fastp software [55] (https://github.com/OpenGene/fastp, accessed on 24 August 2021). Next, the sequence quality was confirmed using Fastp. HISAT2 [56] (http://daehwankimlab.github.io/hisat2, accessed on 24 August 2021) was used to map reads to the Bos taurus genome (version: v96). Mapped reads of IP and input libraries were provided for the R package exomePeak [57] (https://bioconductor.org/packages/exomePeak, accessed on 24 August 2021). This identifies m^6^A peaks using the bed or bigwig formats and is suitable for visualization with IGV software (http://www.igv.org, accessed on 11 January 2022). Motif analysis was conducted with MEME [58] (http://meme-suite.org, accessed on 11 January 2022) and HOMER (http://homer.ucsd.edu/homer/motif, accessed on 11 January 2022). Annotation of called peaks by intersection with gene structure was conducted using the R package ChIPseeker (https://bioconductor.org/packages/ChIPseeker, accessed on 24 August 2021) [59]. The gene assembly and quantification software were StringTie (https://ccb.jhu.edu/software/stringtie, accessed on 24 August 2021) [60], and FPKM was used for quantification. Differential analysis was conducted by using the R package edgeR (https://bioconductor.org/packages/edgeR, accessed on 24 August 2021) [61]. The screening of differentially expressed mRNAs was conducted according to the thresholds of a log_2_ (fold change) >1 or log_2_ (fold change) <−1 and a *p*-value of <0.05. The MeRIP-enriched regions (peaks) were visualized using IGV.

### 4.12. MeRIP-RT-qPCR

All operations were performed using the MeRIP™ m^6^A Kit (cat.A-P-9018, IVDSHOW), following the producer’s instructions. Primer sequences are provided in Supplementary Table S2.

### 4.13. Statistical Analysis

Statistical analysis was performed using GraphPad Prism 8 (GraphPad Software, Inc., San Diego, CA, USA). Data were presented as means ± standard deviations. One-way analysis of variance (ANOVA) and Tukey’s post hoc test was used for the statistical analysis of more than two groups, while the Student’s *t*-test was used for the statistical analysis of two groups. Statistical significance was established when *p* < 0.05. Each experiment was repeated three times.

## 5. Conclusions

In conclusion, our study identified a possible epigenetic mechanism during Cd injury in granulosa cells. Cd induces apoptosis and oxidative stress in granulosa cells by reducing FTO expression to increase the degradation of MNT mRNA through m^6^A modification, thereby decreasing MNT expression. This mechanism may be mediated through the AKT/Nrf2 pathway.

## Data Availability

The data that supports the findings of this study are available from the corresponding author upon reasonable request.

## Supplementary Materials

The following supporting information can be downloaded at: https://www.mdpi.com/article/10.3390/ijms23094948/s1.

## References

[1] Jaffe L.A., Egbert J.R. (2017). Regulation of Mammalian Oocyte Meiosis by Intercellular Communication within the Ovarian Follicle. Annu. Rev. Physiol..

[2] Ahmed M.F., Mokhtar M.B. (2020). Assessing Cadmium and Chromium Concentrations in Drinking Water to Predict Health Risk in Malaysia. Int. J. Environ. Res. Public Health.

[3] Chen H., Teng Y., Lu S., Wang Y., Wang J. (2015). Contamination features and health risk of soil heavy metals in China. Sci. Total Environ..

[4] Ngoc N.T.M., Chuyen N.V., Thao N.T.T., Duc N.Q., Trang N.T.T., Binh N.T.T., Sa H.C., Tran N.B., Ba N.V., Khai N.V. (2020). Chromium, Cadmium, Lead, and Arsenic Concentrations in Water, Vegetables, and Seafood Consumed in a Coastal Area in Northern Vietnam. Environ. Health Insights.

[5] Zhou X., Zheng N., Su C., Wang J., Soyeurt H. (2019). Relationships between Pb, As, Cr, and Cd in individual cows’ milk and milk composition and heavy metal contents in water, silage, and soil. Environ. Pollut..

[6] Hashemi M. (2018). Heavy metal concentrations in bovine tissues (muscle, liver and kidney) and their relationship with heavy metal contents in consumed feed. Ecotoxicol. Environ. Saf..

[7] Suprewicz K., Kozikowska I., Chrobaczyńska-Dylag M., Gał A., Piekarz A., Sikora J., Sławska H., Stawarz R. (2013). Effects of the cigarette smoking on the newborn clinical parametrs and the accumulation of cadmium and lead in the placenta of women from Upper Silesia. Ginekol. Pol..

[8] Richter P.A., Bishop E.E., Wang J., Swahn M.H. (2009). Tobacco smoke exposure and levels of urinary metals in the U.S. youth and adult population: The National Health and Nutrition Examination Survey (NHANES) 1999–2004. Int. J. Environ. Res. Public Health.

[9] Liu J., Luo L.F., Wang D.L., Wang W.X., Zhu J.L., Li Y.C., Chen N.Z., Huang H.L., Zhang W.C. (2019). Cadmium induces ovarian granulosa cell damage by activating PERK-eIF2α-ATF4 through endoplasmic reticulum stress. Biol. Reprod..

[10] Wan X., Zhu J., Zhu Y., Zhu Y., Ma X., Zheng Y., Wang F., Liu Z., Zhang T. (2010). Rat ovarian follicle bioassay reveals adverse effects of cadmium chloride (CdCl2) exposure on follicle development and oocyte maturation. Toxicol. Ind. Health.

[11] Zhang W., Jia H. (2007). Effect and mechanism of cadmium on the progesterone synthesis of ovaries. Toxicology.

[12] Weng S., Wang W., Li Y., Li H., Lu X., Xiao S., Wu T., Xie M., Zhang W. (2014). Continuous cadmium exposure from weaning to maturity induces downregulation of ovarian follicle development-related SCF/c-kit gene expression and the corresponding changes of DNA methylation/microRNA pattern. Toxicol. Lett..

[13] Wang C., Ma W., Su Y. (2013). NF-κB pathway contributes to cadmium-induced apoptosis of porcine granulosa cells. Biol. Trace Elem. Res..

[14] Liaño-Pons J., Arsenian-Henriksson M., León J. (2021). The Multiple Faces of MNT and Its Role as a MYC Modulator. Cancers.

[15] Toyo-oka K., Hirotsune S., Gambello M.J., Zhou Z.Q., Olson L., Rosenfeld M.G., Eisenman R., Hurlin P., Wynshaw-Boris A. (2004). Loss of the Max-interacting protein Mnt in mice results in decreased viability, defective embryonic growth and craniofacial defects: Relevance to Miller-Dieker syndrome. Hum. Mol. Genet..

[16] Hurlin P.J., Zhou Z.Q., Toyo-oka K., Ota S., Walker W.L., Hirotsune S., Wynshaw-Boris A. (2003). Deletion of Mnt leads to disrupted cell cycle control and tumorigenesis. EMBO J..

[17] Hurlin P.J., Quéva C., Eisenman R.N. (1997). Mnt, a novel Max-interacting protein is coexpressed with Myc in proliferating cells and mediates repression at Myc binding sites. Genes Dev..

[18] Meroni G., Reymond A., Alcalay M., Borsani G., Tanigami A., Tonlorenzi R., Lo Nigro C., Messali S., Zollo M., Ledbetter D.H. (1997). Rox, a novel bHLHZip protein expressed in quiescent cells that heterodimerizes with Max, binds a non-canonical E box and acts as a transcriptional repressor. EMBO J..

[19] Eischen C.M., Woo D., Roussel M.F., Cleveland J.L. (2001). Apoptosis triggered by Myc-induced suppression of Bcl-X(L) or Bcl-2 is bypassed during lymphomagenesis. Mol. Cell. Biol..

[20] Jia G., Fu Y., Zhao X., Dai Q., Zheng G., Yang Y., Yi C., Lindahl T., Pan T., Yang Y.G. (2011). N6-methyladenosine in nuclear RNA is a major substrate of the obesity-associated FTO. Nat. Chem. Biol..

[21] Zheng G., Dahl J.A., Niu Y., Fedorcsak P., Huang C.M., Li C.J., Vågbø C.B., Shi Y., Wang W.L., Song S.H. (2013). ALKBH5 is a mammalian RNA demethylase that impacts RNA metabolism and mouse fertility. Mol. Cell.

[22] Rottman F.M., Desrosiers R.C., Friderici K. (1976). Nucleotide methylation patterns in eukaryotic mRNA. Prog. Nucleic Acid Res. Mol. Biol..

[23] Schibler U., Kelley D.E., Perry R.P. (1977). Comparison of methylated sequences in messenger RNA and heterogeneous nuclear RNA from mouse L cells. J. Mol. Biol..

[24] Dominissini D., Moshitch-Moshkovitz S., Schwartz S., Salmon-Divon M., Ungar L., Osenberg S., Cesarkas K., Jacob-Hirsch J., Amariglio N., Kupiec M. (2012). Topology of the human and mouse m6A RNA methylomes revealed by m6A-seq. Nature.

[25] Batista P.J., Molinie B., Wang J., Qu K., Zhang J., Li L., Bouley D.M., Lujan E., Haddad B., Daneshvar K. (2014). m^6^A RNA modification controls cell fate transition in mammalian embryonic stem cells. Cell Stem Cell.

[26] Fu Y., Dominissini D., Rechavi G., He C. (2014). Gene expression regulation mediated through reversible m⁶A RNA methylation. Nat. Rev. Genet..

[27] Wei J., Liu F., Lu Z., Fei Q., Ai Y., He P.C., Shi H., Cui X., Su R., Klungland A. (2018). Differential m^6^A, m^6^A(m), and m^1^A Demethylation Mediated by FTO in the Cell Nucleus and Cytoplasm. Mol. Cell.

[28] Śledź P., Jinek M. (2016). Structural insights into the molecular mechanism of the m^6^A writer complex. eLife.

[29] Shen Z., Liu P., Sun Q., Li Y., Acharya R., Li X., Sun C. (2021). FTO inhibits UPR(mt)-induced apoptosis by activating JAK2/STAT3 pathway and reducing m6A level in adipocytes. Apoptosis Int. J. Program. Cell Death.

[30] Zhao T., Wang J., Wu Y., Han L., Chen J., Wei Y., Shen L., Long C., Wu S., Wei G. (2021). Increased m6A modification of RNA methylation related to the inhibition of demethylase FTO contributes to MEHP-induced Leydig cell injury(☆). Environ. Pollut..

[31] Tang J., Su Q., Guo Z., Zhou J., Zheng F., Yu G., Shao W., Hu H., Wu S., Li H. (2022). N6-methyladenosine(m^6^A) demethylase FTO regulates cellular apoptosis following cobalt-induced oxidative stress. Environ. Pollut..

[32] Zhou L., Han X., Li W., Wang N., Yao L., Zhao Y., Zhang L. (2022). N6-methyladenosine Demethylase FTO Induces the Dysfunctions of Ovarian Granulosa Cells by Upregulating Flotillin 2. Reprod. Sci..

[33] Jiang Z.X., Wang Y.N., Li Z.Y., Dai Z.H., He Y., Chu K., Gu J.Y., Ji Y.X., Sun N.X., Yang F. (2021). The m6A mRNA demethylase FTO in granulosa cells retards FOS-dependent ovarian aging. Cell Death Dis..

[34] Zhao L., Kong X., Zhong W., Wang Y., Li P. (2020). FTO accelerates ovarian cancer cell growth by promoting proliferation, inhibiting apoptosis, and activating autophagy. Pathol. Res. Pract..

[35] Zhao T.X., Wang J.K., Shen L.J., Long C.L., Liu B., Wei Y., Han L.D., Wei Y.X., Wu S.D., Wei G.H. (2020). Increased m6A RNA modification is related to the inhibition of the Nrf2-mediated antioxidant response in di-(2-ethylhexyl) phthalate-induced prepubertal testicular injury. Environ. Pollut..

[36] Liu S., Zhuo L., Wang J., Zhang Q., Li Q., Li G., Yan L., Jin T., Pan T., Sui X. (2020). METTL3 plays multiple functions in biological processes. Am. J. Cancer Res..

[37] Xu G., Liu S., Huang M., Jiang X., Yang M. (2021). Cadmium induces apoptosis of human granulosa cell line KGN via mitochondrial dysfunction-mediated pathways. Ecotoxicol. Environ. Saf..

[38] Zhu M., Miao S., Zhou W., Elnesr S.S., Dong X., Zou X. (2021). MAPK, AKT/FoxO3a and mTOR pathways are involved in cadmium regulating the cell cycle, proliferation and apoptosis of chicken follicular granulosa cells. Ecotoxicol. Environ. Saf..

[39] Ren X., Wang S., Zhang C., Hu X., Zhou L., Li Y., Xu L. (2020). Selenium ameliorates cadmium-induced mouse leydig TM3 cell apoptosis via inhibiting the ROS/JNK /c-jun signaling pathway. Ecotoxicol. Environ. Saf..

[40] Chen X., Wang X., Yang L., Xu H., Wu Y., Wu J., Chen L., Xu C. (2021). Magnesium isoglycyrrhizinate prevents cadmium-induced activation of JNK and apoptotic hepatocyte death by reversing ROS-inactivated PP2A. J. Pharm. Pharmacol..

[41] Yiming Z., Zhaoyi L., Jing L., Jinliang W., Zhiqiang S., Guangliang S., Shu L. (2021). Cadmium induces the thymus apoptosis of pigs through ROS-dependent PTEN/PI3K/AKT signaling pathway. Environ. Sci. Pollut. Res. Int..

[42] Sun Y., Zong C., Liu J., Zeng L., Li Q., Liu Z., Li Y., Zhu J., Li L., Zhang C. (2021). C-myc promotes miR-92a-2-5p transcription in rat ovarian granulosa cells after cadmium exposure. Toxicol. Appl. Pharmacol..

[43] Li L., Zhou M., Chen B., Wang Q., Pan S., Hou Y., Xia J., Zhou X. (2021). ALKBH5 promotes cadmium-induced transformation of human bronchial epithelial cells by regulating PTEN expression in an m6A-dependent manner. Ecotoxicol. Environ. Saf..

[44] Yang H., Li T.W., Ko K.S., Xia M., Lu S.C. (2009). Switch from Mnt-Max to Myc-Max induces p53 and cyclin D1 expression and apoptosis during cholestasis in mouse and human hepatocytes. Hepatology.

[45] Link J.M., Ota S., Zhou Z.Q., Daniel C.J., Sears R.C., Hurlin P.J. (2012). A critical role for Mnt in Myc-driven T-cell proliferation and oncogenesis. Proc. Natl. Acad. Sci. USA.

[46] Du H., Zhao Y., He J., Zhang Y., Xi H., Liu M., Ma J., Wu L. (2016). YTHDF2 destabilizes m^6^A-containing RNA through direct recruitment of the CCR4-NOT deadenylase complex. Nat. Commun..

[47] Bashir N., Shagirtha K., Manoharan V., Miltonprabu S. (2019). The molecular and biochemical insight view of grape seed proanthocyanidins in ameliorating cadmium-induced testes-toxicity in rat model: Implication of PI3K/Akt/Nrf-2 signaling. Biosci. Rep..

[48] Terragni J., Nayak G., Banerjee S., Medrano J.L., Graham J.R., Brennan J.F., Sepulveda S., Cooper G.M. (2011). The E-box binding factors Max/Mnt, MITF, and USF1 act coordinately with FoxO to regulate expression of proapoptotic and cell cycle control genes by phosphatidylinositol 3-kinase/Akt/glycogen synthase kinase 3 signaling. J. Biol. Chem..

[49] Gong Y., Luo S., Fan P., Zhu H., Li Y., Huang W. (2020). Growth hormone activates PI3K/Akt signaling and inhibits ROS accumulation and apoptosis in granulosa cells of patients with polycystic ovary syndrome. Reprod. Biol. Endocrinol. RBE.

[50] Wang S., Lin S., Zhu M., Li C., Chen S., Pu L., Lin J., Cao L., Zhang Y. (2019). Acupuncture Reduces Apoptosis of Granulosa Cells in Rats with Premature Ovarian Failure Via Restoring the PI3K/Akt Signaling Pathway. Int. J. Mol. Sci..

[51] Akino N., Wada-Hiraike O., Terao H., Honjoh H., Isono W., Fu H., Hirano M., Miyamoto Y., Tanikawa M., Harada M. (2018). Activation of Nrf2 might reduce oxidative stress in human granulosa cells. Mol. Cell. Endocrinol..

[52] Khadrawy O., Gebremedhn S., Salilew-Wondim D., Taqi M.O., Neuhoff C., Tholen E., Hoelker M., Schellander K., Tesfaye D. (2019). Endogenous and Exogenous Modulation of Nrf2 Mediated Oxidative Stress Response in Bovine Granulosa Cells: Potential Implication for Ovarian Function. Int. J. Mol. Sci..

[53] Ding H., Zhao J., Liu H., Wang J., Lu W. (2020). BMAL1 knockdown promoted apoptosis and reduced testosterone secretion in TM3 Leydig cell line. Gene.

[54] Wang H.Q., Zhang J.B., Zheng Y., Zhang W.D., Guo H.X., Cong S., Ding Y., Yuan B. (2022). Comprehensive analysis of differences in N6-methyladenosine RNA methylomes in the rat adenohypophysis after GnRH treatment. FASEB J. Off. Publ. Fed. Am. Soc. Exp. Biol..

[55] Chen S., Zhou Y., Chen Y., Gu J. (2018). fastp: An ultra-fast all-in-one FASTQ preprocessor. Bioinformatics.

[56] Kim D., Langmead B., Salzberg S.L. (2015). HISAT: A fast spliced aligner with low memory requirements. Nat. Methods.

[57] Meng J., Lu Z., Liu H., Zhang L., Zhang S., Chen Y., Rao M.K., Huang Y. (2014). A protocol for RNA methylation differential analysis with MeRIP-Seq data and exomePeak R/Bioconductor package. Methods.

[58] Bailey T.L., Boden M., Buske F.A., Frith M., Grant C.E., Clementi L., Ren J., Li W.W., Noble W.S. (2009). MEME SUITE: Tools for motif discovery and searching. Nucleic Acids Res..

[59] Yu G., Wang L.G., He Q.Y. (2015). ChIPseeker: An R/Bioconductor package for ChIP peak annotation, comparison and visualization. Bioinformatics.

[60] Pertea M., Pertea G.M., Antonescu C.M., Chang T.C., Mendell J.T., Salzberg S.L. (2015). StringTie enables improved reconstruction of a transcriptome from RNA-seq reads. Nat. Biotechnol..

[61] Robinson M.D., McCarthy D.J., Smyth G.K. (2010). edgeR: A Bioconductor package for differential expression analysis of digital gene expression data. Bioinformatics.

