# Supplementary material for: FTO Alleviates CdCl2-Induced Apoptosis and Oxidative Stress via the AKT/Nrf2 Pathway in Bovine Granulosa Cells
Source: Int J Mol Sci. 2022 Apr 29;23(9):4948. doi: 10.3390/ijms23094948 (PMC9101166; doi:10.3390/ijms23094948)
Supplement: Supplementary file 1 [file ijms-23-04948-s001.zip › ijms-1696744-supplementary.pdf]

Table S1

siRNA sequences.

siRNA	Sequence(5′→3′)
Negative control	sense: UUCUCCGAACGUGUCACGUTT<br>antisense: ACGUGACACGUUCGGAGAATT
MNT siRNA	sense: GGUGGGCCAGACAGUGCUUTT<br>antisense: AAGCACUGUCUGGCCCACCTT
YTHDF2 siRNA	sense: GCAGUGGGUUCGGUCAUAATT<br>antisense: UUAUGACCGAACCCACUGCTT

Table S2

Primer sequences for real-time qPCR.

Gene	Primer Sequence (5′-3′)	Annealing temp.(°C)	Size (bp)	Genebank No.
β-actin	F: TTGATCTTCATTGTGCTGGGTG<br>R: CTTCCTGGGCATGGAATCCT	54-64	189	NM_173979.3
Bax	F: CGAGTTGATCAGGACCATCAT<br>R: ATGTGGGTGTCCCAAAGTAG	54	168	XM_015458140.2
Bcl2	F: TGGATGACCGAGTACCTGAA<br>R: GAGACAGCCAGGAGAAATCAAA	54	124	NM_001166486.1
Caspase-3	F: TGGCGAAATGCAAAGAACGG<br>R: TGTGAGCGTGCTTTTTCAGC	56	70	NM_001077840.1
YTHDF2	F: GGCAGTGGGTTCGGTCATAA<br>R: ACCGAAGCTTCTCCAAGACG	58	109	NM_001076253.1
ALKBH5	F: TGTCCTTCCAGGCCTAGTGT<br>R: CCCTTCCTAGGGGGATGACA	60	182	NM_001205517.3
FTO	F: CTGCCAGCGTGCCTGAGTTG<br>R: GTACCGTGAAGAGCCTGGTGTTC	63	189	NM_001098142.1
METTL3	F: AGCAGGACTCCACCACAGCAG<br>R: CTTGGTTGGCTCCTTGGCTACTTC	63	92	NM_001102238.2
METTL14	F: TGCCCCGAATTCCTATTTGACTGG<br>R: CACCTCTTCCGCCTCCTCTGG	63	122	NM_001083714.1
SOD	F: AGAGGCATGTTGGAGACCTG<br>R: CAGCGTTGCCAGTCTTTGTA	57	189	NM_174615.2
CAT	F: TGGGACCCAACTATCTCCAG<br>R: AAGTGGGTCCTGTGTTCCAG	57	178	NM_001035386.2
Nrf2	F: CCCAGTCTTCACTGCTCCTC<br>R: TCAGCCAGCTTGTCATTTTG	56	165	NM_001011678.2
NQO1	F: AACCAACAGACCAGCCAATCAGC<br>R: AGCCTCTATGGCAGCCTCCTTC	62	124	NM_001034535.1
BMP4	F: AGAGGGGCTGGAAGAAAAACAG<br>R: GCTCTGGATGGCACTACGG	58	93	NM_001045877.1
MNT	F: GGCTGTTCATGCTCCAAGACA	58	121	NM_001206899.2

	R: GCAGCTCCGTCTCGGGTTT			
VASH1	F: CAGGGAGCTGCAGTACAATCA<br>R: AAATTCCCAGGATCACCGCTT	58	146	NM_001206803.1
TRIM14	F: GCTGAGAAGCTCAAGGCTCAT<br>R: ATTTGCTCTGTGTATGCCTGC	58	155	XM_024996224.1
VCAN	F: GCTTCCCTACCCCTGATAGC<br>R: GTGGGTGAGACAGTTTCTGC	58	112	NM_181035.2
GALNT16	F: CTGCCACCTCCACCTCTGTCTC<br>R: TCCTCCATCTCTGCCTGCCTTC	61	84	NM_001101127.1
WNT5A	F: TTCTCTCCTTCGCCCAGGTT<br>R: AGAGAGGCTGCGCTCCTATG	59	108	NM_001205971.1

Table S3

The information of antibodies used for Western Blot.

Antibodies	Cat NO.	Source	Dilution
Nrf2	AF0639	Affinity,Changchun, China	1：1 000
Cleaved-caspase 3	341034	ZENBIO, Chengdu, China	1：1 000
AKT	R23412	ZENBIO, Chengdu, China	1：1 000
P-AKT	R22961	ZENBIO, Chengdu, China	1：1 000
FTO	R24361	ZENBIO, Chengdu, China	1：1 000
Bcl2	BF9103	Affinity,Changchun, China	1：1 000
Bax	50599-2-lg	ProteinTech, Chicago, IL, USA	1：2 000
MNT	DF4676	Affinity,Changchun, China	1：1 000
β-actin	60008-1-lg	ProteinTech, Rosemont, IL, USA	1：1 0000
Goat Anti-Rabbit IgG	SA00001-2	ProteinTech, Rosemont, IL, USA	1：1 0000
Goat Anti-mouse IgG	SA00001-1	ProteinTech, Rosemont, IL, USA	1：1 0000

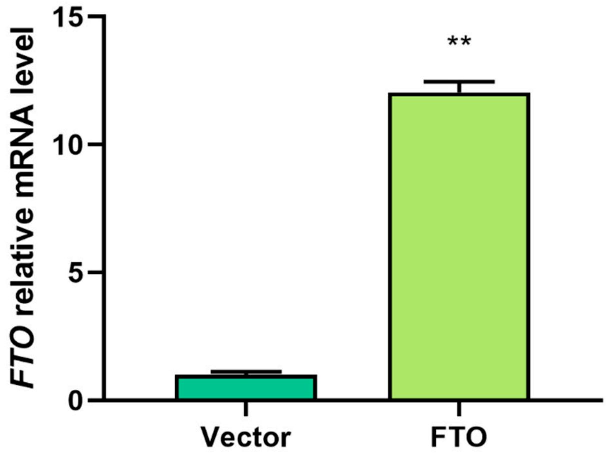

Fig. S1. Analysis of FTO expression levels after transfection pcDNA3.1-FTO or pcDNA3.1-Vector

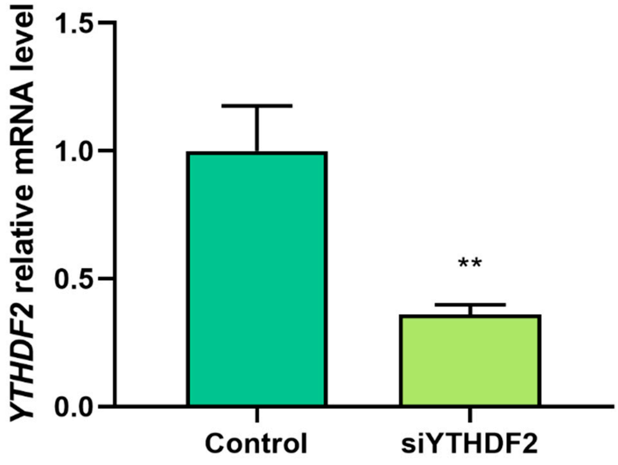

Fig. S2. Analysis of YTHDF2 expression levels after transfection siYTHDF2.

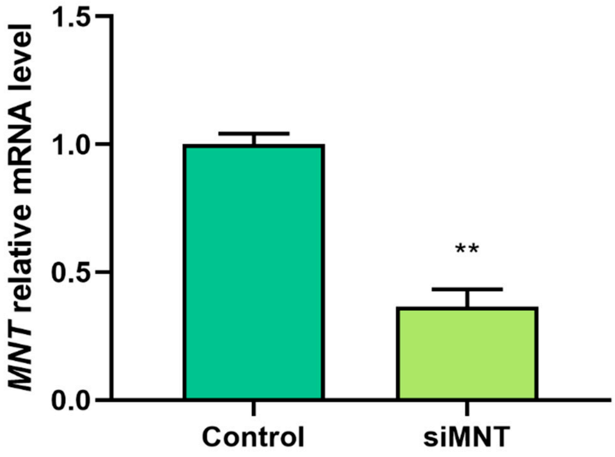

Fig. S3. Analysis of MNT expression levels after transfection siMNT.
